# The classical correlation limits the ability of the measurement-induced average coherence

**DOI:** 10.1038/srep45598

**Published:** 2017-04-04

**Authors:** Jun Zhang, Si-ren Yang, Yang Zhang, Chang-shui Yu

**Affiliations:** 1 College of Mathematics, Institute of Mathematics, Taiyuan University of Technology, Taiyuan 030024, China; 2 School of Physics and Optoelectronic Technology, Dalian University of Technology, Dalian 116024, China

## Abstract

Coherence is the most fundamental quantum feature in quantum mechanics. For a bipartite quantum state, if a measurement is performed on one party, the other party, based on the measurement outcomes, will collapse to a corresponding state with some probability and hence gain the average coherence. It is shown that the average coherence is not less than the coherence of its reduced density matrix. In particular, it is very surprising that the extra average coherence (and the maximal extra average coherence with all the possible measurements taken into account) is upper bounded by the classical correlation of the bipartite state instead of the quantum correlation. We also find the sufficient and necessary condition for the null maximal extra average coherence. Some examples demonstrate the relation and, moreover, show that quantum correlation is neither sufficient nor necessary for the nonzero extra average coherence within a given measurement. In addition, the similar conclusions are drawn for both the basis-dependent and the basis-free coherence measure.

Quantum coherence originating from the quantum superposition principle is the most fundamental quantum feature of quantum mechanics. It plays an important role in various fields such as the thermodynamics^1^,^2^,^3^,^4^,^5^,^6^, the transport theory^7^,^8^,^9^,^10^, the living complexes^11^,^12^,^13^ and so on. With the resource-theoretic understanding of quantum feature in quantum information, the quantification of coherence has attracted increasing interest in recent years^14^,^15^,^16^,^17^,^18^,^19^ and has also led to the operational resource theory of the coherence^20^.

The quantitative theory also makes it possible to understand one type of quantumness (for example, the coherence) by the other type of quantumness such as the entanglement and the quantum correlation, *vice versa*^21^,^22^,^23^,^24^,^25^,^26^,^27^,^28^,^29^,^30^,^31^. For example, for a bipartite pure state, the maximal extra average coherence that one party could gain was shown to be exactly characterized by the concurrence assisted by the local operations and classical communication (LOCC) with the other party^21^. Ref. 22 showed that the maximal average coherence was bounded by some type of quantum correlation in some particular reference framework. In the asymptotic regime, ref. 23 showed that the rate of assisted coherence distillation for pure states was equal to the coherence of assistance under the local quantum-incoherent operations and classical communication. Quite recently, a unified view of quantum correlation and quantum coherence has been given in ref. 24. In addition, if only the incoherent operations are allowed, the state with certain amount of coherence assisted by an incoherent state can be converted to an entangled state with the same amount of entanglement^32^ or a quantum-correlated state with the same amount of quantum correlation^33^.

In this paper, *instead of the quantum correlation*, we find, it is *the classical correlation* of a bipartite quantum state that limits the extra average coherence at one side induced by the unilateral measurement at the other side. We also find the necessary and sufficient condition for the zero maximal average coherence that could be gained with all the possible measurements taken into account. Besides, we show, through some examples, that quantum correlation is neither sufficient nor necessary for the extra average coherence subject to a given measurement. We have selected both the basis-dependent and the basis-free coherence measure to study this question and obtain the similar conclusions. In particular, one should note that all our results are valid for the positive-operator-valued measurement (POVM), even though we only consider the local projective measurement in the main text.

## Results

### The upper bound on the extra measurement-induced average coherence

#### Coherence measure

To begin with, let’s first give a brief review of the measure of the quantum coherence^14^. If a quantum state 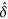 can be written as


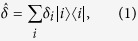



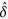
 is incoherent with respect to the basis {|*i*〉}. Let 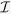 denotes the set of incoherent states, then the operator 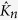 is the incoherent operation if it satisfies 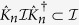. Thus a good coherence measure *C(ρ*) of a *d*-dimensional state *ρ* should be:

(p1) Nonnegative-i.e., *C(ρ*) ≥ 0 and *C(ρ*) = 0 if and only if the quantum state *ρ* is incoherent.

(p2) Monotonic-i.e., 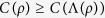 for any incoherent operation 
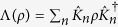
; and strongly monotonic if 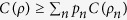 with 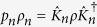.

(p3) Convex-i.e., 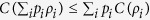.

Even though there are many good coherence measures such as the coherence measures based on *l*_1_-norm, trace norm, fidelity, the relative entropy and so on^14^,^15^,^16^,^17^,^18^,^19^, in this paper we will only employ the relative entropy to quantify the quantum coherence, i.e.,


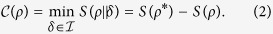


where 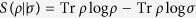 is the relative entropy, 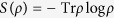 is the von Neumann entropy and 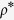 is the diagonal matrix by deleting all the off-diagonal entries of any *ρ* (we will use this notation throughout the paper). For simplicity, we will restrict ourselves in the computational basis throughout the paper. In contrast, the basis-free coherence (or the total coherence)^34^ is quantified by


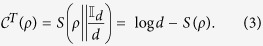


Note that 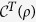 quantifies the maximal coherence of a state with all the bases taken into account.

#### The Classical correlation as the upper bound

**Figure 1:**
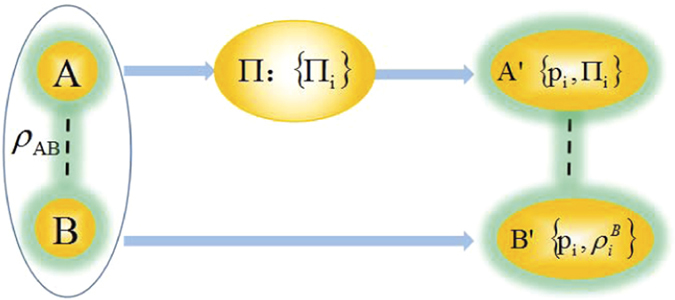
Illustration of the two-player game on the measurement-induced average coherence.

Now let’s turn to our game sketched in Fig. 1. Suppose two players, Alice and Bob, share a two-particle quantum state *ρ*_*AB*_ and Alice performs some projective measurement 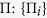 on her particle and sends her outcomes to Bob. Bob isn’t allowed to do any operation. Based on Alice’s outcomes, Bob will obtain the state 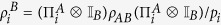 with the probability 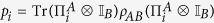. Thus in the computational basis, the measurement-induced average coherence (MIAC: Bob’s average coherence induced by Alice’s measurement 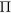) is given by


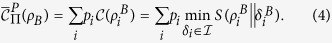


Similarly, the measurement-induced average total coherence (MIATC: Bob’s average total coherence induced by Alice’s measurement 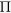) is


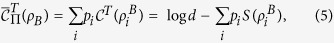


with *d* denoting the dimension of Bob’s space. With Alice’s measurement 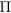, the Bob’s average coherence is usually different from the coherence of 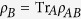. The extra MIAC 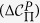 and the extra MIATC 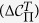 can be defined as


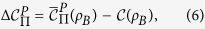



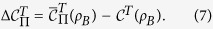


It is obvious that 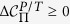 which is impied by the convexity of the coherence 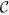, that is, 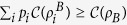 with 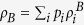.

Thus our main results can be given by the following theorems.

**Theorem 1:** For a bipartite quantum state *ρ*_*AB*_, the extra MIAC 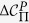 is not greater than the extra 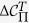, i.e.,


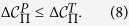


*Proof*. Based on Eq. (4), we have


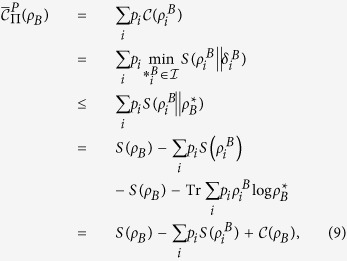


with 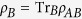. Substituting the definition of MIATC (Eq. (5)) into Eq. (9), we can obtain the


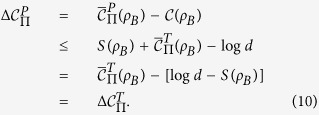


The inequality holds if all Bob’s states *ρ*_*B*_ and 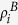 have the same diagonal entries. The proof is completed. 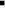


**Theorem 2:** For a bipartite quantum state *ρ*_*AB*_, the extra MIAC 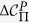 is upper bounded by the classical correlation of *ρ*_*AB*_, that is,


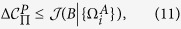


where the classical correlation is defined by


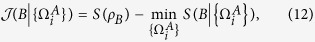


with 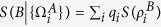 and 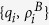 defined by


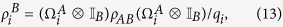


and the corresponding probability


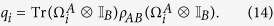


Eq. (11) saturates if 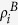 induced by the measurement 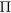 achieves the classical correlation 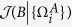 and 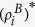
’s are the same for all *i*. An example is the pure state 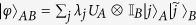 where *U*_*A*_ is unitary, |*j*〉, 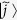 are the local computational basis.

**Theorem 3:** The extra MIATC 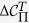 for a bipartite quantum state *ρ*_*AB*_ is upper bounded by the classical correlation 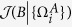 of *ρ*_*AB*_, i.e.,


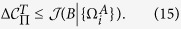


The equality holds for the pure *ρ*_*AB*_.

*Proof*. From the classical correlation, we have


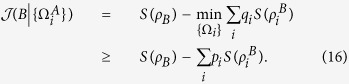


Substituting Eq. (5) into Eq. (16), one can arrive at


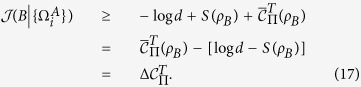


Since both 
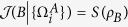
 and 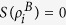 hold for pure *ρ*_*AB*_^35^, the inequality (16) saturates for the pure quantum state *ρ*_*AB*_. The proof is finished. 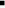


All the above three theorems hold for any projective measurement, so if we specify the particular measurement such that the maximal extra MIAC or MIATC can be achieved, the three theorems are also valid, which can be given in a rigorous way as:

**Corollary 1.** For a bipartite state *ρ*_*AB*_ with the reduced density matrix *ρ*_*B*_, the maximal extra MIAC and the maximal extra MIATC satisfy


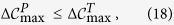


and


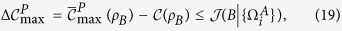



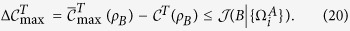


with


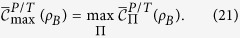


If *ρ*_*B*_ is incoherent, we have


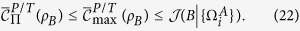


*Proof*: It is obvious from theorem 1, 2 and 3. 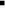


**Corollary 2:** If *ρ*_*AB*_ satisfies 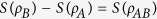, then


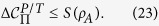


*Proof*. If the initial quantum state *ρ*_*AB*_ satisfies the 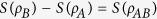, we have^36^


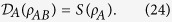


where 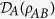 is the quantum discord defined by 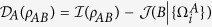 with 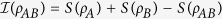. Thus one can easily show 
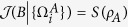
 which completes the proof. 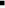


**Theorem 4:** Taking all Alice’s possible measurements into account, no extra MIAC is present if and only if the state *ρ*_*AB*_ is block-diagonal under Bob’s computational basis or a product state.

*Proof*. Consider the computational basis {|*i*〉_*B*_}, the state 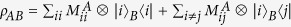 where 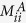 is Hermitian and positive and 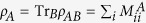. It is obvious that if 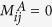 for all 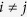, the states Bob obtains are always diagonal subject to {|*i*〉_*B*_}. That is, no extra MIAC can be obtained. If *ρ*_*AB*_ is a product state which implies 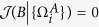, it means that the upper bound of the extra MIAC is zero based on Theorem 2. So no extra MIAC could be obtained.

On the contrary, no extra MIAC includes two cases: one is that the final average coherence is zero, and the other is that the final nonzero average coherence is not increased compared with the coherence of 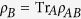. The first case means that Alice performs a measurement 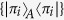 (optimal for the maximal average coherence) such that Bob obtains an ensemble 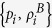 where 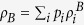 with all 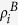 diagonal. Thus *ρ*_*AB*_ can be written as


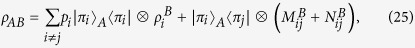


where 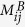 is diagonal and 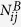 has no nonzero diagonal entries. Assume there is at least one nonzero matrix 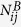 among all *i, j*, then one can always select a projector 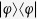 such that 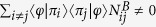. This means that Bob can get a state with some coherence. In other words, 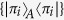 is not the optimal measurement, which is a contradiction. So we have 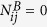. Under this condition, one can find from Eq. (25) that *ρ*_*AB*_ is block-diagonal subject to Bob’s basis {|*i*〉_*B*_}. The second case implies that there exists a decomposition 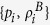 (optimal for the maximal average coherence) with 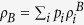 such that 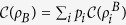 which, however, is only satisfied when all 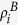 are the same for nonzero 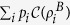, since 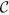 is a convex function. Thus we have 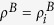 which leads to 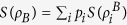. Now we claim that 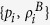 is also optimal for the classical correlation. This can be seen as follows. If there exists another decomposition 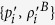 for the classical correlation, 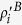 cannot be the same, which will lead to the larger average coherence due to the convexity of 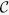. This is a contradiction. So 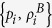 is the optimal decomposition for the classical correlation, that is, 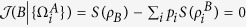 which implies *ρ*_*AB*_ is a product state. The proof is finished. 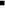


**Theorem 5:** Consider all Alice’s possible measurements, no extra MIATC is present if and only if the state *ρ*_*AB*_ is a product state.

*Proof*. A product state has no classical correlation, i.e., 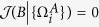 which implies that the upper bound of the extra MIATC is zero in terms of Theorem 3. Thus no extra MIATC could be obtained.

On the contrary, no extra MIATC implies that 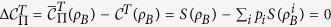, namely 
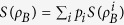
. Similar to the proof of theorem 4, one can find that 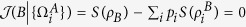 which corresponds to a product state *ρ*_*AB*_. The proof is finished. 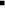


#### Examples

The above theorems mainly show that, even though the coherence is the quantum feature of a quantum system, in the particular game as sketched in Fig. 1, the extra average coherence obtained by Bob with the assistance of Alice’s measurement is well bounded by the classical correlation of their shared state, instead of the quantum correlation. However, one can find that the necessity for all the attainable bounds is to share the pure states which happen to own the equal quantum and classical correlations. Therefore, one could think that the classical correlation is trivial in contrast to the quantum correlation (e.g., quantum correlation serves as a tight upper bound, but is less than classical correlation). The following examples show that it is not the case.

*Example 1. The extra average coherence could be induced in classical*-*classical states*. Suppose a bipartite state is given by


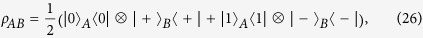


with 
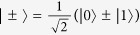
, the reduced quantum state 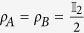 is incoherent. So the classical correlation is equal to the total correlation, i.e.,


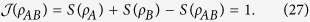


If the subsystem *A* is measured by the projective measurements 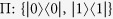, subsystem B will collapse to the state 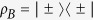 with the probability 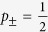. The extra MIAC and the extra MIATC subject to the measurement 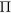 can be calculated as


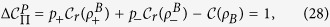



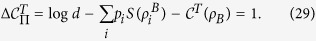


If the subsystem *A* is measured by the projective measurement 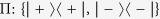, subsystem B will collapse to the state 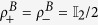 with the equal probability. So there is no extra MIAC and MIATC. This example shows that the extra average coherence is well bounded by the classical correlation. In particular, it also shows that the extra average coherence could exist even though not any quantum correlation is present.

*Example 2. No extra average coherence could be induced in the classical*-*quantum state*. Set the classical-quantum state as


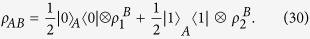


with 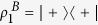 and 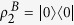. The reduced quantum states are given by


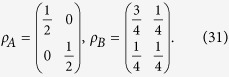


Since there is no quantum correlation subject to subsystem A, the corresponding classical correlation is directly determined by the total correlation as


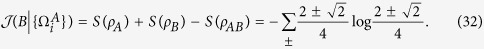


Suppose that the projective measurement 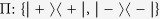 is performed on subsystem A while the subsystem B will collapse on the state 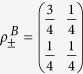 with the equal probability 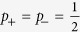. It is obvious that there is no extra average coherence 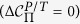 gained by this measurement. However, if the projective measurement is selected as 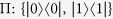, subsystem B will be at the state 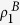 and 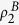 with the equal probability. Therefore, the *nonzero* extra average coherence can be obtained as


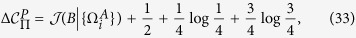



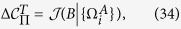


with 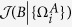 given by Eq. (32). This example shows that an improper measurement could induce no extra average coherence even though quantum correlation is absent.

*Example 3. No extra average coherence could be induced in the quantum*-*classical state*. Suppose the quantum-classical state is given by


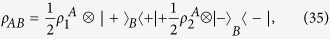


with 
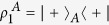
 and 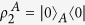. It is easy to see that the reduced quantum state 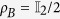 is incoherent, i.e., 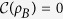. The classical correlation is


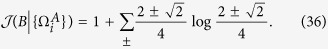


If the projective measurement 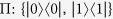 is used on subsystem A, subsystem B will be on the states 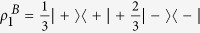 and 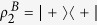 with the corresponding probability 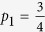 and 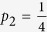. Thus a simple calculation can show


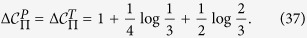


However, if we select another projective measurement 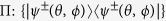 where 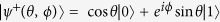 and 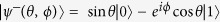 with cot2 *θ* = cos *ϕ*, subsystem B will collapse to


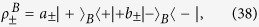


where 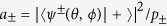 and 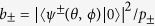 with the probability 
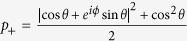
 and 
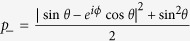
. It is easy to demonstrate that *a*_±_ = *b*_±_ for cot2 *θ* = cos *ϕ* which further leads to 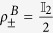. Thus there is no extra average coherence can be gained in terms of this measurement constraint, that is,


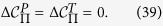


Similar to the second example, an improper measurement could induce no extra average coherence even though quantum correlation is present.

*Example 4. The classical correlation can be tighter than the quantum correlation*. Consider a Bell-diagonal state


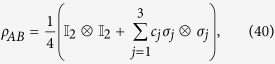


where 
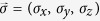
 is the Pauli matrices. *ρ*_*AB*_ is symmetric under exchanging the subsystems. The classical and the quantum correlations are respectively given by ref. 37


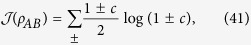



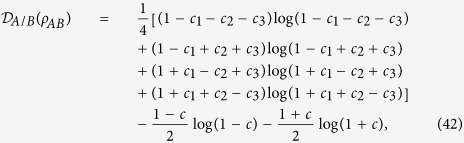


with *c* = max{|*c*_1_|, |*c*_2_|, |*c*_3_|}.

Suppose the projective measurement 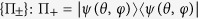, 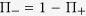 with 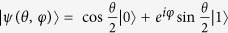 is performed on subsystem A, subsystem B will collapse, with the equal probability 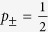, on the states


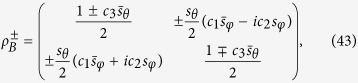


where *s*_*x*_ = sin(*x*) and 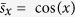. In addition, it is obvious that the reduced quantum states 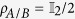 which implies 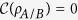. So the extra average coherence can be directly given by the MIAC or MIATC as


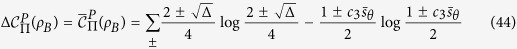



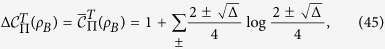


with 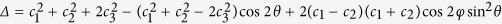.

**Figure 2:**
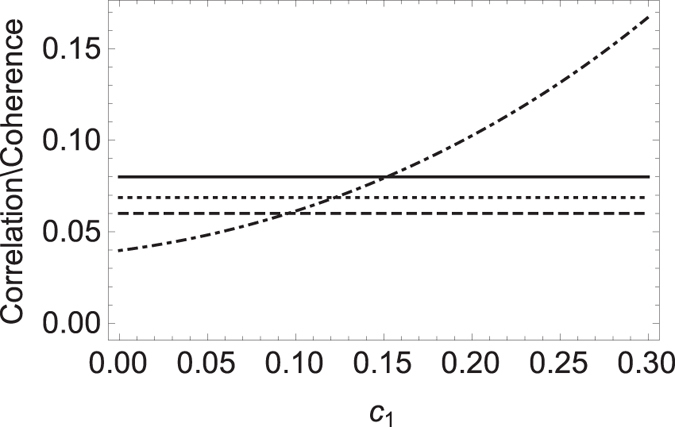
The classical correlation 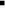 (solid line), the quantum correlation 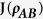 (dotted-dashed line), the (extra) MIATC 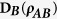 (dotted line) and the (extra) MIAC 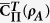 (dashed line) versus *c*_1_ for the Bell-diagonal state.

In Fig. 2, we plot the quantum and classical correlations and the extra average coherence with the varying *c*_1_. The parameters are chosen as *θ* = 2*π*/3, 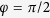 and *c*_2_ = 0.33, *c*_3_ = 0.22. The solid line, dotted-dashed line, dotted line and dashed line correspond to the classical correlation, the quantum correlation, the MIATC and the MIAC, respectively. One can find that the classical correlation serves as the good upper bound for both the (extra) MIATC and the (extra) MIAC and meanwhile, the (extra) MIATC is always greater than the (extra) MIATC. However, the quantum correlation crossing the classical correlation, the (extra) MIATC and the (extra) MIAC with the increasing *c*_1_ cannot act as a good bound.

## Discussion

Before the end, we would like to emphasize that all the results in the paper are valid for the POVMs, since it was shown^38^ that the classical correlations always attained by the rank-one POVM. In addition, we have claimed that Bob isn’t allowed to do any operation, which is mainly for the basis-dependent coherence measure. In fact, when we consider the basis-free coherence measure, it is equivalent to allowing Bob to select the optimal unitary operations on his particle. In this case, theorem 3 implies that for pure states the extra MIATC is the exact quantum entanglement of their shared state (von Neumann entropy of the reduced density matrix). Thus the coherence also provides an operational meaning for the pure-state entanglement under LOCC.

To sum up, we employ the basis-dependent and basis-free coherence measure to study the extra average coherence induced by a unilateral quantum measurement. Despite that the coherence is the most fundamental quantum feature, we find that the extra average coherence is limited by the classical correlation instead of the quantum correlation. In addition, we find the necessary and sufficient condition for the zero maximal average coherence. We also show that the quantum correlation is neither sufficient nor necessary for the extra average coherence by some examples.

## Methods

### Proof of Theorem 2

We will give the main proof the theorem 2. in the main text. Following Eq. (9), we have


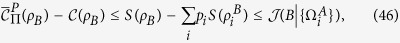


where the second inequality holds due to the optimal 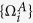 implied in Eq. (12). So Eq. (11) is satisfied.

In addition, Eq. (11) saturates if both Eqs (9) and (15) saturate. Eq. (15) means that 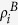 induced by the measurement 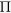 achieves the classical correlation 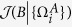 and Eq. (9) implies 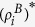
’s are the same for all *i*. In order to find an explicit example, suppose 
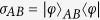
 with


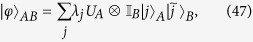


with the real *λ*_*j*_ satisfying 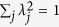. It is obvious 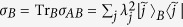 is incoherent with respect to the basis 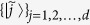. It means


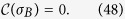


In order to select a proper measurement, Alice first applies a unitary operation 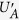 such that


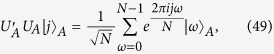


with *N* denoting the dimension of the subsystem A. Thus 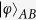 becomes


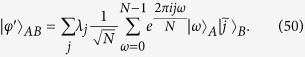


Now Alice performs the projective measurement 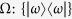 on 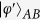, Bob will obtain his state as


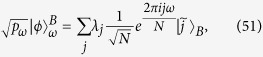


with the probability *p*_*ω*_ corresponding to the measurement outcome *ω*. Bob’s MIAC can be given by


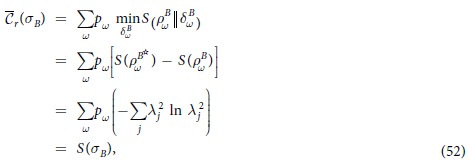


with 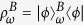. For the pure state 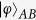, it can prove that the classical correlation 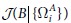 is exactly given by


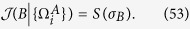


Eqs (48), (52) and (53) show that Eq. (11) saturates for the pure state given by Eq. (47). The proof is finished. 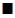


## Additional Information

**How to cite this article:** Zhang, J. *et al*. The classical correlation limits the ability of the measurement-induced average coherence. *Sci. Rep.* **7**, 45598; doi: 10.1038/srep45598 (2017).

**Publisher's note:** Springer Nature remains neutral with regard to jurisdictional claims in published maps and institutional affiliations.
